# Sequential activation of ERα-AMPKα signaling by the flavonoid baicalin down-regulates viral HNF-dependent HBV replication

**DOI:** 10.1038/s41401-024-01408-3

**Published:** 2024-10-30

**Authors:** Yi-jun Niu, Cheng-jie Xia, Xin Ai, Wei-ming Xu, Xiao-tong Lin, Ying-qi Zhu, Hai-yan Zhu, Xian Zeng, Zhong-lian Cao, Wei Zhou, Hai Huang, Xun-long Shi

**Affiliations:** 1 Department of Biological Medicines & Shanghai Engineering Research Center of Immunotherapeutics, Fudan University School of Pharmacy, Shanghai, 201203 China; 2 Department of Chemistry, Fudan University, Shanghai, 201203 China

**Keywords:** antiviral flavonoid, baicalin, hepatocyte nuclear factor, hepatitis B virus, ERα, AMPKα

## Abstract

Baicalin (BA), a natural component found in many traditional Chinese medicines, exerts protective effects against several viruses. Although our previous studies have revealed that the anti-hepatitis B virus (anti-HBV) activity of BA depends on hepatocyte nuclear factor (HNF) signaling, the specific mechanisms remain unclear. The present study explored the potential signaling mechanisms involved in BA-mediated HBV suppression. Transcriptomic analysis suggested that BA significantly modulates the estrogen receptor (ER) and AMPK signaling pathways in HepG2 cells. The ER alpha (ERα) binding affinity of BA and its estrogen-like agonist activity were subsequently verified through molecular docking assays, BA-ERα affinity detection experiments, ERα luciferase reporter gene assays, and qRT-PCR. ERα knockdown (shRNA) and AMPK inhibition (Compound C and doxorubicin [Dox]) experiments revealed that the sequential activation of the ERα-LKB1-AMPK-HNF signaling axis is essential for the anti-HBV effects of BA. This study indicates that BA may trigger the ERα-AMPKα-HNF pathway to inhibit HBV replication, providing insights into its potential protective mechanisms against other viruses.

## Introduction

Viral hepatitis B, caused by the hepatitis B virus (HBV), is among the most common infectious diseases in the world. While the incidence of HBV infection has gradually declined owing to the increased adoption of hepatitis B vaccination, the WHO estimates that in 2022, approximately 254 million people worldwide were living with chronic hepatitis B infection, with 1.2 million new infections occurring every year [1]. Hepatitis B also accounted for an estimated 1.1 million deaths globally, primarily due to cirrhosis and hepatocellular carcinoma (primary liver cancer) [1].

Currently, the first-line clinical treatment for HBV infection involves the administration of nucleoside analogs (e.g., tenofovir and entecavir) [2] and PEG-interferon preparations [3]. However, the effectiveness of these treatments is limited. Therefore, new anti-HBV drugs with novel mechanisms of action that can enhance the therapeutic effects of known anti-HBV drugs and prevent drug resistance are urgently warranted. One potential candidate is baicalin (BA), a natural flavonoid extracted from *Scutellaria baicalensis* that exhibits various pharmacological properties, including anti-inflammatory [4], anti-oxidant, and anticancer activities [5]. A recent study highlighted the antibacterial efficacy of BA-based dynamic covalent hydrogels, demonstrating the utility of BA in a range of therapeutic contexts [6]. Such findings underscore the therapeutic potential of BA, particularly with respect to its anti-HBV properties, calling for additional research into the molecular mechanisms underlying its inhibitory effects on HBV replication.

Evidence from previous studies shows that BA can comprehensively inhibit HBV DNA replication, RNA production, and virus antigen (HBeAg and HBsAg) secretion in HepG2.2.15 cells and pHBV-transfected HepG2 cells. Moreover, it can effectively reverse drug resistance in HBV and exert synergistic therapeutic effects in combination with entecavir, likely via the hepatocyte nuclear factor 4α (HNF4α)-HNF1α axis [7]. However, it is unlikely that BA directly binds to HNFs, since these molecules typically act as downstream transcription factors and not receptors. Therefore, the mechanisms through which BA regulates HNFs remain to be elucidated.

HBV is a sex hormone-reactive virus. Epidemiological investigations show that the incidence of HBV infection and HBV-associated cirrhosis and hepatic carcinoma is significantly higher among men than among women [8]. This sex-related difference has been linked to the activation of estrogen receptors (ERs) by the high levels of estrogen in women. Estrogen (17β-estradiol) has been found to promote the heterodimerization of ERα and HNF4α by activating ERα, competitively reducing HNF4α dimerization and the binding of HNF4α to the HBV enhancer I (Enh I), thereby inhibiting HBV transcription [9].

In non-HBV pharmacological models (e.g., breast cancer cells, osteogenesis models, and angiogenesis models), BA has shown both inhibitory and excitatory effects on ERs [10–13]. Our previous studies have confirmed that BA can down-regulate HBV RNA in HepG2.2.15 cells while also decreasing the expression of HNF1α and HNF4α [14]. In addition, BA can promote the heterodimerization of ERα-HNF4α in HepG2 cells [7], which suggests that ERα could be crucial for the BA-mediated inhibition of covalently closed circle DNA (cccDNA) transcription in HBV.

Therefore, in this study, we explored the mechanistic links between ERα and HNFs that may mediate the anti-HBV effects of BA.

## Materials and methods

### Reagents and materials

BA (98% HPLC purity) was purchased from ChromaBio (Chengdu, China). BA was dissolved in DMSO (40 mg/mL) and stored at 4 °C. Before use, the BA stock solution was diluted using cell culture medium and sterilized using a 0.22-μm filter. Dox, compound C, tamoxifen, and metformin were procured from Meilunbio (Dalian, China). Fulvestrant was purchased from Beyotime Biotechnology (Shanghai, China). 17β-estradiol was supplied by Sigma-Aldrich (St. Louis, USA).

### Cell culture and transfection

HepG2 cells were cultured in Dulbecco’s Modified Eagle's Medium (DMEM) supplemented with 10% fetal bovine serum (FBS) (Gibco, Waltham, Australia) at 37 °C in 5% CO_2_. HepG2 cells were transfected with the pHBV1.2 plasmid using Lipo6000™ Transfection Reagent (Beyotime Biotechnology) according to the manufacturer’s instructions. The pHBV1.2-transfected HepG2 cells were called “pHBV1.2-HepG2” cells [7]. The pHBV1.2 plasmid — which contained a 1.2-fold HBV DNA genome (genotype C, subtype Adr) — was gifted by Prof. Zheng-hong Yuan from Fudan University, China.

The ERα gene was knocked down in HepG2 cells using an shRNA lentiviral vector (LV), which was purchased from GeneChem (Shanghai, China). All protocols were carried out according to the manufacturer’s instructions.

### HBV infection and treatment in mice

Male and female BALB/c mice (age: 5–6 weeks, weight: 18–22 g) were purchased from SLACCAS (SPF II Certificate. No. SCXK2012-0002, Shanghai, China). The mice were maintained in plastic cages at 23 ± 2 °C under a relative humidity of 50% ± 10%, with free access to food and water.

Each BALB/c mouse was hydrodynamically injected with 10 μg of the pHBV1.2 plasmid (dissolved in 2 mL of PBS) via the tail vein [15]. The next day, the infected mice were randomly divided into four groups and treated with the relevant treatment agents (blank control, 10 mg/kg BA, 20 mg/kg BA, and 40 mg/kg BA; intragastric administration once a day). Seven days after infection, the mice were sacrificed, and retro-orbital blood samples were collected and centrifuged. The serum was used for ELISA and RT-PCR analysis. Liver tissue was isolated to examine hepatic injury, and a liver tissue homogenate was prepared to extract RNA and proteins for the subsequent analysis of signaling pathway activity.

All animal experiments were performed in accordance with the “Guidelines for the Care and Use of Medical Laboratory Animals” (Ministry of Health of the People’s Republic of China, 1998), and the study was approved by the Ethics Committee of Fudan University (Shanghai, China) (approval No. 2015-O3-HC-SXL-01).

### Detection of HBeAg and HBsAg in vivo and in vitro

In vitro: HBsAg and HBeAg were detected using the ELISA method. The kits were purchased from Kehua Bioengineering Co., Ltd., Shanghai, China. pHBV1.2-HepG2 cells were incubated with BA (0–100 μM), and supernatants were collected every 2 days for the ELISA-based quantification of HBeAg and HBsAg.

In vivo: Blood collected from mice was centrifuged to collect serum. The serum was diluted 2000-fold, and the levels of HBeAg and HBsAg were detected using an ELISA kit.

### Transcriptomics analysis

The pHBV1.2-HepG2 cells were treated with 100 μM BA for 96 h. The cell samples were used for mRNA isolation, library preparation, and sequencing by the Majorbio Bio-pharm Technology Corporation (Shanghai, China). Differentially expressed genes were subjected to Gene Ontology (GO) and Kyoto Genes and Genomes (KEGG) pathway enrichment analysis via the free online Majorbio Cloud Platform (https://www.majorbio.com). The NCBI accession number is PRJNA 799795.

### Molecular docking

Two PDB files of the three-dimensional (3D) structure of ERα (1GWR and 3ERT) were selected for docking analysis and downloaded from the Protein Data Bank (https://www.rcsb.org/). 1GWR was combined with the natural agonist E2, and 3ERT was combined with antagonists. These complexes were preprocessed before Glide Docking using the protein preparation wizard of the Maestro10.2 program by Schrodinger. Meanwhile, the compound was prepared using the Maestro ligand preparation wizard. The Glide Docking module (Glide 5.8) in Maestro 10.2 was used to dock the compound into the binding site of both receptors. The compound was subjected to a Monte Carlo Multiple Minimum conformational search using the OPLS_2005 force field, and the most reasonable conformation was selected. In addition, another molecular docking software, AutoDock 4.2, was used along with the visualization software Pymol 2.4 (Schrodinger) to dock BA with the 3ERT and 1GWR regions of ERα. Based on the results of molecular docking, the free energy of binding between the compound and the protein and the estimated *K*_i_ value were predicted. The experimental *K*_i_ value was obtained from the PDB database.

### Surface plasmon resonance (SPR) analysis of the kinetic constants of BA against ERα

The SPR technique was applied to characterize the kinetic constants of BA against human ERα (ab82606, Abcam, London, UK) using a Biacore T200 instrument (GE Healthcare, Chicago, USA). Experiments were performed at 25 °C. ERα was immobilized onto the CM5 sensor chip surface using the Amine Coupling kit. BA was serially diluted two-fold from 58.9 μM to 0.12 μM using the running buffer (HBS-EP^+^). Then, the mixtures were injected at a flow rate of 30 μL/min, with an association time of 45 s and a dissociation time of 60 s. The surface was then regenerated with HBS-EP^+^ buffer for 30 s at a flow rate of 30 μL/min.

The kinetic data were processed with Evaluation Software version 3.0. The data were fitted globally to a 1:1 binding model. The association *K*_a_ (M^−1^·s^−1^) and dissociation *K*_d_ (s^−1^) kinetic rate constants were obtained. The equilibrium dissociation constant *K*_D_ (M) was determined based on the equation *K*_D_ = *K*_d_/*K*_a_.

All the reagents and kits were purchased from GE Healthcare.

### ERE luciferase reporter gene experiment

The pHBV1.2 plasmid was transfected into HepG2 cells using the Lipo6000™ transfection reagent. The cells were then transfected with the ERE luciferase reporter plasmid (11528ES03; Yeasen Biotechnology, Shanghai, China) and ESR1-expressing plasmid (constructed by Fenghui Biotechnology, Beijing, China) using the Lipo6000™ Transfection Reagent for 12 h. This was followed by treatment with BA alone or BA + tamoxifen (100 nM) for 12 h. E2 (10 nM) was used as the positive control. The cells were pretreated with reagents from a luciferase reporter gene assay kit (Yeasen Biotechnology) according to the manufacturer’s instructions. The relative light unit (RLU) values were detected with an automatic fluorescence chemiluminescence analyzer (Fluoroskan Ascent FL, Thermo Scientific, Waltham, USA).

### Gene transcription analysis

Total RNA was isolated from HepG2 cells using the TRIzol reagent and reverse transcribed to cDNA using the PrimeScript™ RT reagent kit with gDNA Eraser (TaKaRa Bio, Beijing, China), as previously reported [16]. qRT-PCR was performed to analyze gene transcription using the SYBR Premix Ex Taq™ (TaKaRa Biomedical Technology, Beijing, China) and StepOne Plus Real-Time PCR System (Thermo Scientific). The following thermocycling parameters were applied: 95 °C for 15 min, followed by 40 cycles of 95 °C for 15 s and 60 °C for 1 min. The RQ values were calculated using the ^ΔΔ^CT method. GAPDH was used as the internal reference (Table 1).

**Table 1:** Primers used for RT-PCR.

Primer name	Sequence (5′-3′)	
GAPDH	Forward	CATGTTCGTCATGGGGTGAACCA
	Reverse	AGTGATGGCATGGACTGTGGTCAT
HNF1α	Forward	CCTGTCCCAACACCTCAACAA
	Reverse	TTGAAACGGTTCCTCCGC
HBV pgRNA	Forward	CTCAATCTCGGGAATCTCAATGT
	Reverse	TGGATAAAACCTAGCAGGCATAAT
^a^Total HBV-specific transcripts	Forward	ATCCTGCTGCTATGCCTCATCTT
	Reverse	ACAGTGGGGGAAAGCCCTACGAA
HBV-DNA	Forward	TCACCAGCACCATGCAAC
	Reverse	AAGCCACCCAAGGCACAG
hB1F	Forward	GGCTTATGTGCAAAATGGCAGATC
	Reverse	GCTCACTCCAGCAGTTCTGAAG
pS2	Forward	CCAGGCCCAGGAAGAAACAT
	Reverse	AACAGCAACCTCTCTCCGTG
C/EBPα	Forward	TGGACAAGAACAGCAACGAGTA
	Reverse	ATTGTCACTGGTCAGCTCCAG
Hepcidin	Forward	CTCCTTCGCCTCTGGAACAT
	Reverse	AGTGGCTCTGTTTTCCCACA
CyclinD1	Forward	TGTTCGTGGCCTCTAAGATGAAG
	Reverse	AGGTTCCACTTGAGCTTGTTCAC

^a^Total HBV-specific transcripts: Designed based on all RNA molecules transcribed in HBV.

### Western blot

The culture medium was discarded from the culture flasks and plates, and the cells were washed with PBS. Western and immunoprecipitation lysis buffer (Beyotime Biotechnology) was added to the cells, and the supernatant was collected after centrifugation (12,000 × *g* for 15 min). Subsequently, a BCA detection kit (Beyotime Biotechnology) was used to measure protein concentrations. Protein samples were separated using SDS-PAGE and transferred to polyvinylidene fluoride (PVDF) membranes (Thermo Scientific). PVDF membranes were then blocked with Western blocking solution (Beyotime Biotechnology) for 1 h. Subsequently, the membranes were incubated overnight with the indicated primary antibodies (1:1000 dilution) on a shaker at 4 °C. The membranes were then incubated with the secondary antibody (1:1000) on a shaker at room temperature (25 °C) for 1.5 h. The obtained bands were visualized via chemiluminescence reactions (ECL, Merck Millipore, Birica, USA), and gray values were analyzed using Image J software (NIH, Bethesda, Rockville, USA).

Antibodies against ERα, p-ERα, p-p38MAPK, C/EBPα, AMPKα, p-AMPKα, LKB1, p-LKB1, β-actin, and GAPDH were obtained from Cell Signaling Technology (Danvers, USA). The antibody against ERβ was purchased from Wanlei Biotechnology (Shenyang, China).

### Cellular ATP and AMP measurement

The treated HepG2 cells were washed with PBS, lysed on ice using a lysis buffer for 10 min, and centrifuged at 4 °C and 12,000 × *g* for 5 min to obtain the supernatant. The cellular ATP and AMP levels in the supernatant were detected using an ATP detection kit (Beyotime Biotechnology) and AMP detection kit (Fantaibio, Shanghai, China) according to the manufacturer’s instructions. For ATP detection, chemiluminescence was determined using an automatic fluorescence chemiluminescence analyzer. Meanwhile, AMP content was measured using a microplate reader at *A*_450 nm_. The ATP and AMP concentrations of the samples were calculated according to standard curves.

### Statistical analysis

Data were presented as the mean ± standard deviation. All experiments were repeated at least thrice. The obtained data were analyzed using Student’s *t*-tests or one-way ANOVA followed by Bonferroni’s test. The associations between HBeAg and HBsAg levels were analyzed using Pearson’s correlation coefficient. *P* < 0.05 was considered statistically significant. Statistical analyses were performed using GraphPad Prism 8 (La Jolla, USA).

## Results

### Activation of ERα may reduce HBV infection efficiency

**Fig. 1:**
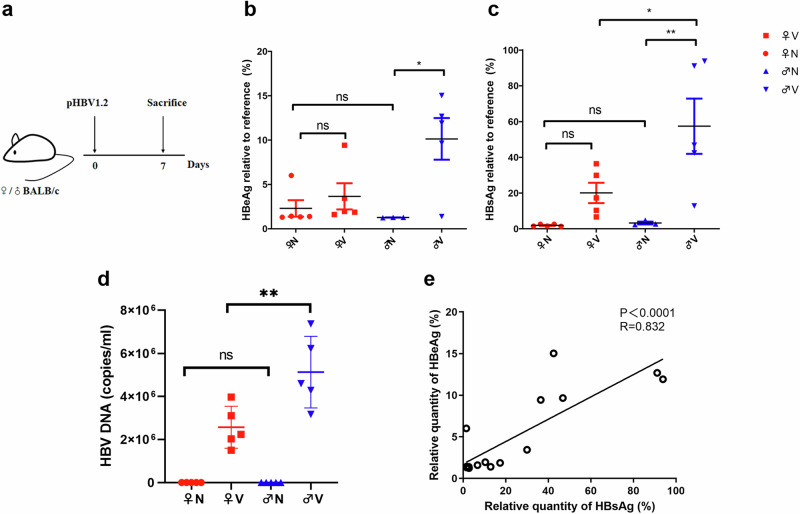
The influence of sex on HBV infection. **a** Experimental paradigm for the detection of HBV antigen levels. Levels of serum HBeAg (**b**), HBsAg (**c**), and HBV-DNA (**d**) in male and female mice after 7 days of HBV infection. **e** Pearson’s correlation analysis of HBeAg and HBsAg levels. All data are presented as the mean ± SD, *n* = 5; *P < 0.05, **P < 0.01 vs. control. ns not significant.

Several reports have shown that estrogen exerts anti-HBV effects in vitro and in vivo [17]. In order to evaluate the influence of sex on HBV infection, male and female BALB/c mice were used to construct an acute HBV infection model and sacrificed at 7 days post-infection (Fig. 1a). Subsequently, the levels of serum HBV antigens (HBsAg and HBeAg) and HBV-DNA were detected. As shown in Fig. 1b–d, the HBsAg, HBeAg, and HBV-DNA levels in female mice were significantly lower than those in male mice. This confirmed the sex differences in HBV infection, suggesting that some substances or activated targets in females may exert anti-HBV effects. Pearson’s correlation coefficient analysis revealed a linear correlation between HBsAg and HBeAg levels (Fig. 1e). Given that ERα is highly expressed and activated in female mice [18], it was possible that ERα could be the key factor inhibiting HBV replication in these mice. To minimize the influence of host estrogen, male mice were used for subsequent experiments to explore the role of ERα in the anti-HBV effects of BA.

### BA can exert estrogen-like agonist activity by binding to ERs

**Fig. 2:**
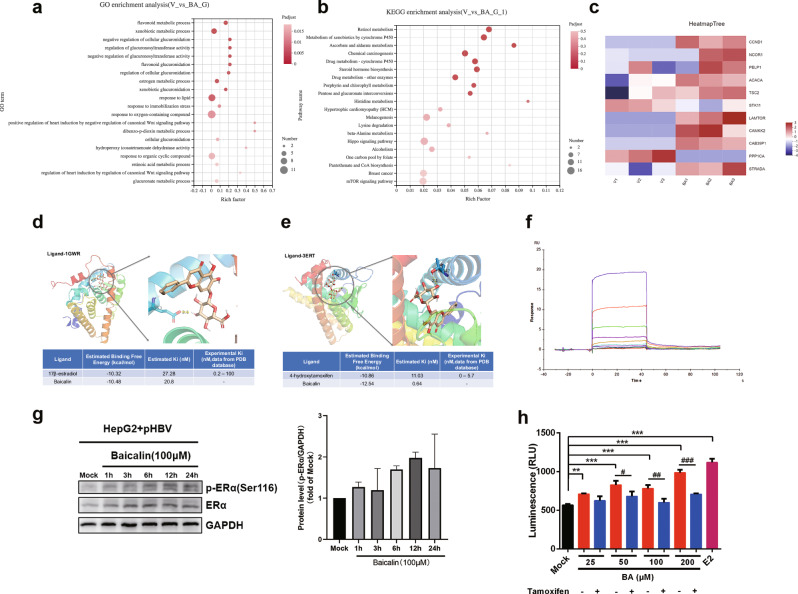
Baicalin (BA) can bind to ERα and demonstrate estrogen-like activity. **a**, **b** GO and KEGG enrichment analysis (virus control and BA). **c** Heat maps of genes differentially expressed between the BA and virus control groups. **d**, **e** Molecular docking of BA and ERα. **f** Affinity between BA and the ERα protein, and a sensorgram of ERα–BA interactions. **g** Western blot-based detection of ERα and p-ERα levels in pHBV1.2-transfected HepG2 cells treated with 100 μM BA. **h** Luciferase reporter gene assay. HepG2 cells were transfected with the pHBV1.2 plasmid for 6 h. Then, they were transfected with the ERE luciferase reporter plasmid and ESR1-expressing plasmid for 12 h. This was followed by treatment with BA or BA + tamoxifen (100 nM) for 12 h. E2 (50 nM) served as the positive control. The relative light unit (RLU) values of each sample were measured to estimate the activation of ERα.

The transcriptomic changes in BA-treated cells were detected using RNA-sequencing technology, and the differentially expressed genes were analyzed for GO and KEGG enrichment. The results revealed that BA up-regulated ER and AMPK signaling pathways in HepG2 cells (vs. control) (Fig. 2a, b). As shown in Fig. 2c, specific ER-related genes (CCND1, NCOR1, and PELP1) were significantly up-regulated in the BA-treated cells, suggesting that BA can exert estrogen-like agonist activity, consistent with previous findings [19–21]. Furthermore, an increase in the expression of AMPK-related genes (ACACA, TSC2, STK11, LAMTOR, CAMKK2, CAB39P1, and PPP1CA) was also observed in BA-treated cells [22–28].

Molecular docking simulations indicated that BA may bind to the 3ERT region of ERα (Fig. 2d). The two hydroxyl groups of BA and the hydroxyl oxygen on the ASP351 residue of ERα appeared to form hydrogen bonds (distances of 2.7 Å and 2.8 Å, respectively). Another hydroxyl group of BA was observed to form a hydrogen bond with the hydroxyl oxygen on the THR347 residue (distance of 3.1 Å). Further, the hydroxyl group on the flavonoid ring of BA could form a hydrogen bond with the VAL534 residue. As shown in Fig. 2e, BA could also bind to the 1GWR region of ERα by forming a hydrogen bond with the GLU353 residue (distance of 2.1 Å). This affinity between BA and ERα was also confirmed by the kinetic constants for their interaction (*K*_a_ 1.408 × 10^3^ M^−1^·s^−1^, *K*_d_ 1.358 × 10^−2^ s^−1^, and *K*_D_ 9.640 × 10^−6^ M) (Fig. 2f). Western blot analysis further confirmed that BA enhances ERα activation by increasing its phosphorylation (Fig. 2g).

Moreover, we constructed an ERE over-expression luciferase reporter and ESR1 cell line. Notably, in this cell line, BA promoted the activation of the ERE element in a dose-dependent manner. Additionally, this effect was inhibited by tamoxifen, a known antiestrogen drug (Fig. 2h).

These results revealed that BA could bind to ERα and exhibit estrogen-like agonist activity.

### BA inhibits HBV replication in an ERα-dependent manner

**Fig. 3:**
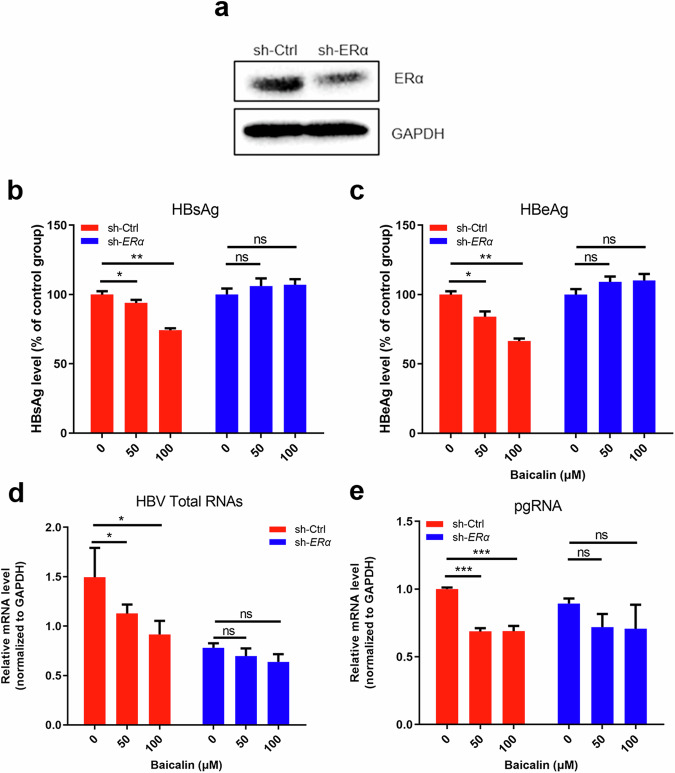
ERα knockdown abolishes the anti-HBV effects of BA. **a** Stable knockdown of ERα. HepG2 cells were transfected with a lentivirus (LV) containing a mock sequence (pMCS-IRES-EGFP) (sh-Ctrl) or a human ERα shRNA-expressing cassette (sh-ERα). ERα and GAPDH levels were detected using Western blot analysis. **b**, **c** HBsAg and HBeAg levels in sh-Ctrl and sh-ERα HepG2 cells after transfection with the pHBV1.2 plasmid and treatment with baicalin (0, 50, and 100 μM) for 4 days. HBsAg and HBeAg were detected using ELISA. *n* = 3; *P < 0.05 and **P < 0.01 vs. virus control. ns, not significant. **d**, **e** Relative mRNA levels of pgRNA and total HBV-specific transcripts in the sh-Ctrl and sh-ERα HepG2 cells after transfection with the pHBV1.2 plasmid and treatment with baicalin (0, 50, and 100 μM) for 4 days. pgRNA and total HBV-specific transcripts were detected using qRT-PCR. *n* = 3; *P < 0.05, **P < 0.01, and ***P < 0.001 vs. virus control. ns not significant.

To further confirm the role of ERα in the effects of BA, the ERα gene was knocked down in HepG2 cells using an shRNA lentiviral vector (Fig. 3a). As shown in Fig. 3b–e, ERα knockdown completely abolished the inhibitory effects of BA against the secretion of HBV antigens (HBsAg and HBeAg) and synthesis of HBV RNAs (total RNAs and pgRNA). This indicated that ERα may play a key role in the anti-HBV therapeutic effects of BA.

### BA sequentially activates the ERα-LKB1-AMPKα axis

**Fig. 4:**
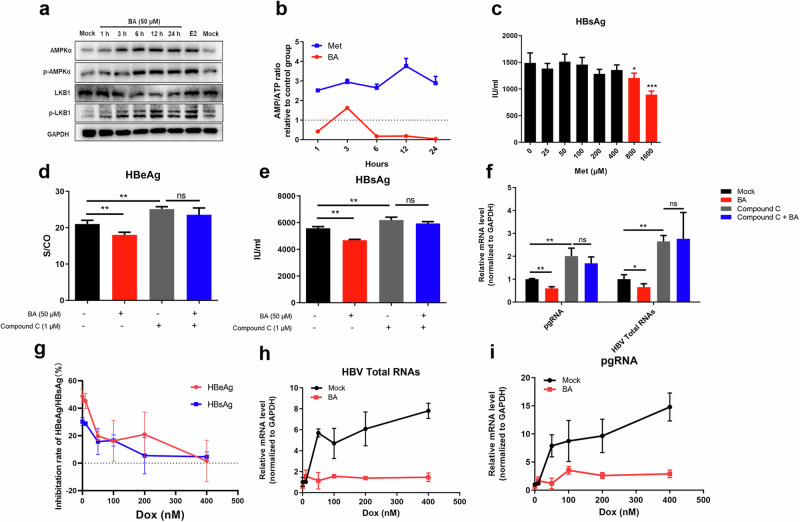
The anti-HBV effects of baicalin (BA) are mediated by the ERα-LKB1-AMPKα axis. **a** Effect of BA on the protein expression levels of AMPKα, p-AMPKα, LKB1, and p-LKB1 in pHBV1.2-HepG2 cells. HepG2 cells were transfected with pHBV1.2 and treated with 50 μM BA for 1, 3, 6, 12, and 24 h or with 10 nM E2 for 24 h. The protein expression of AMPKα, p-AMPKα, LKB1, and p-LKB1 in pHBV1.2-HepG2 cells was detected using Western blot analysis. **b** Effect of BA or metformin (Met) on the AMP/ATP ratio in pHBV1.2-HepG2 cells. HepG2 cells were transfected with pHBV1.2 and treated with BA (50 μM) or Met (50 μM) for 1, 3, 6, 12, and 24 h. The cellular contents of ATP and AMP were measured using ATP and AMP detection kits. The AMP/ATP ratio was calculated and normalized based on the value in the control group without BA or Met treatment. *n* = 3. **c** Effect of Met on HBsAg levels in pHBV1.2-HepG2 cells. HepG2 cells were transfected with pHBV1.2 and treated with Met (0–1000 μM) for 48 h. HBsAg levels in the culture supernatant were detected using ELISA. *n* = 4; *P < 0.05 and ***P < 0.001 vs. control. **d**, **e** Effect of Compound C on HBsAg and HBeAg levels in pHBV1.2-HepG2 cells treated with BA. HBsAg and HBeAg levels in the culture supernatant were detected using ELISA. *n* = 4; **P < 0.01 vs. control. ns, not significant. **f** Effect of Compound C on the relative mRNA levels of pgRNA and total HBV-specific transcript levels in pHBV1.2-HepG2 cells treated with BA. Total HBV-specific transcripts and pgRNA were quantified using qRT-PCR. *P < 0.05 and **P < 0.01 vs. control. *n* = 3. **g**–**i** Effect of doxorubicin (Dox) on HBeAg/HBsAg levels and the relative mRNA levels of pgRNA and total HBV-specific transcripts in pHBV1.2-HepG2 cells treated with BA. HepG2 cells were transfected with pHBV1.2 and treated with Dox (0–400 nM) or Dox (0–400 nM) + BA (100 μM) for 48 h. HBeAg and HBsAg levels were detected using ELISA, and HBV total RNA and pgRNA levels were quantified using qRT-PCR. *n* = 3.

ERα was previously reported to activate AMPKα through the LKB1 protein kinase [29]. In our experiments, the phosphorylation-based activation of AMPKα and LKB1 was also found to be enhanced in BA-treated cells, and this enhancement appeared time-dependent (Fig. 4a).

As AMPKα activation may be influenced by the increase in intracellular AMP/ATP [30], the influence of BA on intracellular AMP/ATP was evaluated. In these experiments, metformin (Met) was used as the positive control. As shown in Fig. 4b, c, in contrast to BA treatment, metformin treatment significantly up-regulated the AMP/ATP ratio, but it weakly reduced HBsAg levels. This suggested that BA-induced AMPKα activation was not linked to the cellular AMP/ATP ratio but could be driven by the LKB1-AMPKα pathway.

Compound C (an AMPKα inhibitor) was further used to identify the role of AMPKα in the anti-HBV effects of BA. As shown in Fig. 4d–f, compound C completely attenuated the inhibitory effects of BA on the secretion of HBV antigens (HBeAg and HBsAg) and synthesis of intracellular HBV-related RNAs. Similar results were observed when AMPKα was inhibited using another inhibitor, Dox. Dox treatment reversed the BA-induced inhibition of HBeAg and HBsAg (Fig. 4g). As shown in Fig. 4h, i, the intracellular levels of total HBV RNA and pgRNA were enhanced after Dox mono-treatment and reversed after co-treatment with BA and Dox.

The above data collectively suggested that BA may inhibit HBV transcription by activating the ERα-LKB1-AMPKα axis.

### BA down-regulates hepatocyte nuclear factors (HNF1α and C/EBPα) via the ERα-LKB1-AMPKα axis

In our previous study, we reported that BA down-regulates HBV transcription in an HNF1α-dependent manner [7]. Here, we examined whether BA regulates HNF1α signaling via the sequential activation of the ERα-LKB1-AMPKα axis.

**Fig. 5:**
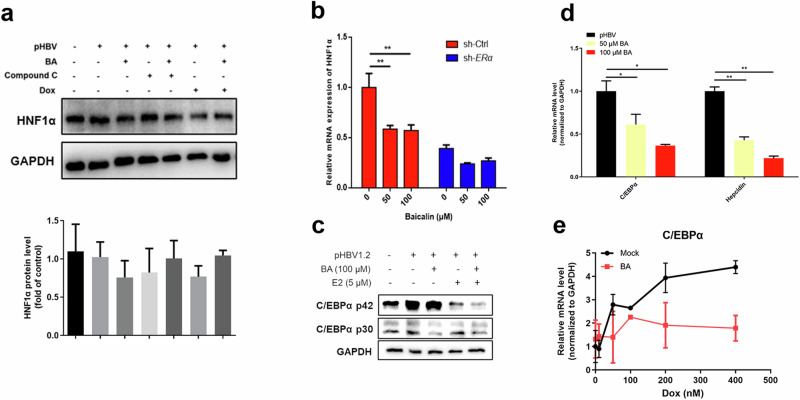
Baicalin (BA) down-regulates HNF1α and C/EBPα. **a** pHBV1.2-HepG2 cells were treated with BA (50 μM), compound C (10 μM), and Dox (50 nM) for 12 h. The protein expression of HNF1α was detected using Western blot analysis. *n* = 3. **b** shERα-HepG2 cells were transfected with pHBV1.2 for 6 h and treated with BA (0, 50, and 100 μM) for 4 days. HNF1α transcription was detected using qRT-PCR. **P < 0.01 vs. control. ns not significant. *n* = 3. **c** pHBV1.2-HepG2 cells were treated with 50 μM BA and 5 μM E2 (single and combined treatment) for 48 h. The expression of CEBPα p42 and p30 was detected using Western blot analysis. **d** pHBV1.2-HepG2 cells were treated with 50 μM and 100 μM BA. The transcription of C/EBPα and its downstream gene Hepcidin was analyzed using qRT-PCR. **e** pHBV1.2-HepG2 cells were treated with Dox (0–400 nM) alone or Dox (0–400 nM) + BA (100 μM) for 48 h. C/EBPα mRNA levels were detected using qRT-PCR. *n* = 3.

pHBV1.2-hepG2 cells were treated with BA, E2, and AMPKα inhibitors (Dox and compound C) in various combinations, and the effects on HNF1α expression were examined. As illustrated in Fig. 5a, HNF1α was down-regulated after treatment with BA alone. However, the effects of BA were reversed after co-treatment with Dox and compound C. In addition, when ERα was knocked down via shRNA (Fig. 5b), the BA-mediated suppression of HNF1α was attenuated. These results proved that the BA-induced down-regulation of HNF1α was dependent on the activation of ER and AMPKα.

The lipid metabolism-related gene C/EBPα is down-regulated after AMPKα activation [31, 32]. Recent studies have shown that C/EBPα is a hepatic nuclear factor and regulates HBV nuclear transcription by binding to the HBV enhancer and other liver nuclear factors [33]. Therefore, we explored the changes in C/EBPα levels in BA-treated cells. As shown in Fig. 5d, BA effectively down-regulated the transcription of C/EBPα and its downstream target gene Hepcidin. Compared with E2, BA notably reduced the expression of C/EBPα p30 (Fig. 5c). As expected, AMPKα inhibition (Dox treatment) increased the transcription levels of C/EBPα, but this effect was reversed by co-treatment with BA (Fig. 5e).

Thus, our results indicated that BA suppresses HBV transcription through the ERα-AMPKα-HNF pathway.

### GPER does not influence the anti-HBV effects of BA

Previous studies have shown that E2 may down-regulate the expression of HNF1α through the HNF4α-HNF1α axis by activating ERα [9]. Some studies have also reported that although fulvestrant (ICI182780) significantly inhibits intracellular ERα, it activates the membrane receptor of estrogen, i.e., G protein-coupled ER (GPER, also known as GPR30) [34, 35].

**Fig. 6:**
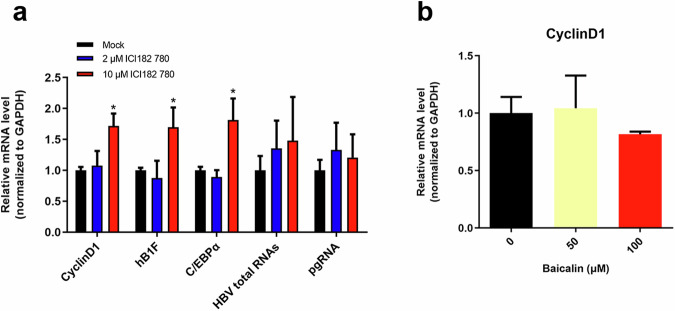
Influence of fulvestrant on the transcription of CyclinD1 and HBV. **a**, **b** pHBV1.2-HepG2 cells were treated with 2 μM or 10 μM fulvestrant and BA (0, 50, or 100 μM) for 48 h. The levels of C/EBPα mRNA, CyclinD1 mRNA, HBV total RNAs, and pgRNA were detected using qRT-PCR.

To examine the role of GPER in the anti-HBV effects of BA, pHBV-HepG2 cells were treated with 2 μM or 10 μM fulvestrant for 48 h. Transcription analysis (Fig. 6a) showed that 10 μM fulvestrant significantly increased the transcription of C/EBPα and the GPER target gene CyclinD1, suggesting that fulvestrant also activated GPER. However, fulvestrant treatment did not influence HBV RNA production (Fig. 6a). Moreover, CyclinD1 levels were not altered in BA-treated cells (Fig. 6b), indicating that the anti-HBV effects of BA depend on the activation of ERα, but are independent of GPER.

## Discussion

ERs, as relatively upstream transcription factors, mediate a variety of regulatory processes, including inflammation, autophagy, and cell proliferation [36]. BA, a plant estrogen, has been found to exhibit a range of pharmacologic effects [4, 5]. In this study, male mice appeared more susceptible to HBV infection than female mice, indicating that the activation of ERs plays an important role in HBV infection. These findings suggested that the anti-HBV effects of BA may depend on ER activation. Subsequent molecular docking analysis revealed that BA could bind to the 1GWR and 3ERT regions of ERα. Notably, the 1GWR region (binding site of estrogen) mainly mediates ERα activation, while the 3ERT region (binding site of selective ER modulators) exerts an inhibitory effect [37]. Moreover, the findings showed that BA can significantly enhance the phosphorylation of ERα, and that shERα can abolish the inhibitory effects of BA against HBV, indicating that BA suppresses HBV replication in an ERα-dependent manner. In the future, a more detailed understanding of the specific BA and ERα binding sites that mediate the anti-HBV action of BA could be obtained through site-mutation analyses.

Activated ERs can activate AMPKα by directly binding to its βγ subunit binding region as ERα homodimers or ERα-ERβ heterodimers or by interacting with LKB1 kinase [29]. This study revealed that BA can activate AMPKα through an ERα-dependent pathway, thereby down-regulating HBV transcription and replication. Moreover, the effects were reversed after AMPKα inhibition (compound C and Dox). An increase in the cellular AMP/ATP ratio was found to activate AMPKα. However, metformin (an agonist of AMPKα) markedly increased the AMP/ATP ratio in pHBV-HepG2 cells while mildly inhibiting HBV antigen secretion. Interestingly, only a slight increase in the AMP/ATP ratio was observed in BA-treated cells, indicating that BA mainly activates AMPKα via the ERα-LKB1 axis.

Our previous study elucidated the crucial role of HNF1α in the anti-HBV effects of BA. Here, we further demonstrated that HNF1α is regulated by ERα and AMPKα. When pHBV-hepG2 cells were co-treated with AMPKα inhibitors or an ERα inhibitor, the down-regulation of HNF1α expression induced by BA was reversed. Given that previous findings show that the HNF4-HNF1α axis is inhibited after AMPKα activation [38], we speculated that BA may sequentially activate ERα-LKB1-AMPKα signaling to inhibit HBV replication in an HNF1α-dependent manner.

Interestingly, the transcription of the lipid metabolism-related gene C/EBPα was found to be inhibited by BA in this study, and this effect was mediated by AMPKα activation. Studies show that sterol regulatory element-binding protein 1c (SREBP-1c) is a direct phosphorylation target of AMPK, and the phosphorylation of its Ser372 residue by AMPK can block its maturation [31]. Proteolytic cleavage, which inhibits hepatic steatosis and adipogenesis, down-regulates a series of lipogenesis-related genes, including C/EBPα [39]. These findings could explain the lipid-lowering effect of BA observed in previous studies. The present study showed that BA can significantly down-regulate the p30 isoform of C/EBPα, but has little effect on the p42 isoform. Whether the down-regulation of C/EBPα is involved in the anti-HBV effects of BA thus needs to be verified in future experiments through C/EBPα knockdown. Besides, although the relevant molecular mechanisms were elucidated in this study through in vitro experiments, the findings remain to be validated in vivo.

Notably, the effect of AMPKα inhibitors on the anti-HBV effect of BA could be related to AMPKα-induced autophagic behavior. Autophagy is believed to promote the intracellular replication of HBV. The activation of PRKAA, the catalytic subunit of AMPKα, can stimulate the degradation of autophagolysosomes to ultimately suppress HBV replication [40]. The inhibitory effects of BA on autophagy have been reported in our previous study on the anti-influenza virus effects of BA [41]. However, whether autophagy is involved in the anti-HBV effects of BA still needs to be explored.

Polyphenols, including BA, are known for their diverse biological activities, including their antiviral and virucidal effects [42]. Tannins, a subtype of polyphenols, have garnered considerable attention for their potential to combat viral infections. Recent studies have highlighted the role of natural tannins as potent anti-SARS-CoV-2 compounds, demonstrating their ability to inhibit viral replication [43]. For example, research on tannins such as procyanidin condensed tannins derived from *Mitragyna speciosa* (Kratom) has demonstrated their potential value as virucidal agents against SARS-CoV-2 [44], further underscoring the antiviral capabilities of polyphenols. While BA itself is a flavonoid, the literature illustrates the broader importance of polyphenols as valuable compounds in antiviral research, creating new avenues for treatments against viral pathogens. This growing body of evidence supports the exploration of polyphenols, such as BA, not only for their direct antiviral effects but also for their potential role in modulating the immune responses and cellular processes that affect viral replication.

In summary, in this study, we identified a new mechanism through which BA suppresses HBV infection by activating ERs and inhibiting nuclear factors in hepatocytes. This study provides new experimental insights and methodological ideas for antiviral research on phytoestrogens and other traditional Chinese medicines. Moreover, given the estrogen-mimicking activity of BA, we report that BA can activate AMPKα and thereby provide anti-HBV effects, which could explain the various pharmacological effects of this compound.
